# Regional and institutional origin of articles in Journal of Minimal Access Surgery

**DOI:** 10.4103/0972-9941.58505

**Published:** 2009

**Authors:** Sudhir Kumar Thakur, Shalabh Gupta, Shashank R Gupta, Somen Jha

**Affiliations:** Department of Surgery, Santosh Medical College and Hospital, Ghaziabad - 201 001, UP, India

Dear Editor,

The geographical distribution of publications as an indicator of the research productivity of individual countries, regions or Institutions has become a field of interest. A few studies on geographical distribution of articles in peer reviewed indexed journals have been conducted to identify the countries that are making maximum contributions to the development of medical science.[1] Ever since the first laparoscopic cholecystectomy was done by Erich Mühe in 1985, minimal access surgery (MAS) has witnessed a phenomenal growth. When the first hesitant laparoscopic cholecystectomy was done in India in early 1990, surgical activists came down heavily on proponents of this patient friendly surgery on the grounds that it was inappropriate for poor countries.[2] However, the criticism did not hamper the growth of (MAS) in India. Publication in peer reviewed good journals is one of the major indices to judge the growth of any particular field as well as research rating of any geographical area and Institution. We tried to find out the current status of minimal access surgery in India by taking into account regional and Institutional contributions of articles in (JMAS). The official website of the journal was the source of all inputs.[3] All the articles except the editorial ones were included in the study. Twentyfour countries have contributed articles to this journal so far. The study was focussed on the articles of Indian origin. A total of 149 articles were studied, out of these 80 were of Indian origin. The contribution from Maharashtra, Delhi and West Bengal taken together was 69%. Mumbai contributed 24 articles, Delhi 13 and Kolkata 10. As far as the Institutes were concerned Sir Ganga Ram Hospital with eight articles had maximum publications. Although 11 States contributed to the journal, the majority of the articles were mainly from three states viz, Maharashtra, Delhi and West Bengal. The articles from Mumbai were not only maximum but also had even contributions from different Institutes. Mumbai was the first city in India to adopt Minimal Access Surgery. Dr. T. E. Udwadia was the first to perform laparoscopic cholecystectomy in 1990. The maximum contributions from this city are a true reflection of his pioneering endeavours. Among the Institutions, Sir Ganga Ram Hospital, was on the top followed by ILS Multi Speciality Clinic, Salt Lake, Kolkata. It is a well established fact that Sir Ganga Ram Hospital has been the hub of activities related to MAS in the north eastern region of India. Contributions from all undergraduate (M.B.B.S.) teaching hospitals taken together was 30%. Even the Govt Medical Colleges were not lagging behind in their efforts. The three metropolitan cities, which adopted the skills early, had better representation in the journal. The conclusion is that India has truly adopted MAS and is using it extensively and successfully. Tracking multiple articles by the same authors and Institutions covering same, similar or different research topics or projects could also prove useful in identifying evolving research trends, centres of expertise, existing collaboration nuclei (and potential collaborators) in different research areas from worldwide. It would be interesting to conduct the study after 5 years when one can have a significant sample size.
